# The Utility of Symptom Association Probability (SAP) in Predicting Outcome After Laparoscopic Fundoplication in Patients with Abnormal Esophageal Acid Exposure

**DOI:** 10.1007/s11605-023-05753-2

**Published:** 2023-07-05

**Authors:** Donata Vaiciunaite, Sven E. Eriksson, Inanc S. Sarici, Ping Zheng, Ali H. Zaidi, Blair Jobe, Shahin Ayazi

**Affiliations:** 1 Esophageal Institute, Department of Surgery, Allegheny Health Network, Pittsburgh, PA USA; 2 Department of Surgery, Drexel University, Philadelphia, PA USA

**Keywords:** Antireflux surgery, Nissen fundoplication, Symptom association probability (SAP), GERD, Outcome

## Abstract

**Introduction:**

Abnormal DeMeester score on pH monitoring is a well-established predictor of favorable outcome after antireflux surgery (ARS). Esophageal pH monitoring also facilitates analysis of the temporal association between symptoms and reflux episodes. This association can be expressed with several symptom–reflux association indices with symptom association probability (SAP) being the most reliable. SAP is often used as an adjunct to DeMeester score during preoperative assessment of patients seeking ARS. However, data on the utility of SAP in predicting ARS outcome is limited. The aim of this study was to determine the utility of SAP as an adjunct to DeMeester score in predicting outcomes after fundoplication.

**Methods:**

Records of patients who underwent primary fundoplication from 2015 to 2021 were reviewed. Patients with a preoperative DeMeester score >14.7 on Bravo pH monitoring were included. A SAP >95% was considered SAP-positive. Favorable outcome was defined as freedom from proton pump inhibitors (PPIs) and patient satisfaction at 1 year postoperatively. Outcomes were compared based on the presence and number of SAP-positive symptoms, individual typical and atypical SAP-positive symptoms, and within demographic, clinical, and reflux severity subgroups.

**Results:**

The final study population consisted of 597 patients (71.4% female) with a median (IQR) age of 59.0 (49–67). At a mean (SD) follow-up of 10.5 (8) months, 82.0% patients achieved favorable outcome (satisfaction and freedom from PPI), freedom from PPI was 91.7%, and satisfaction was 87.4%. SAP was positive in 430 (72.0%) patients, of which 221 (37.0%) had one SAP-positive symptom, 164 (27.5%) had two SAP-positive symptoms, and 45 (7.5%) had all three SAP-positive symptoms. There was no association between having at least one SAP-positive symptom and favorable outcome (*p*=0.767). There was no difference in favorable outcome between patients with one, two, or all SAP-positive symptoms (0.785). Outcomes were comparable for SAP-positive typical (*p*=0.873) and atypical symptoms (*p*=1.000) and all individual symptoms (*p*>0.05). Outcomes were also comparable within all subgroups (*p*>0.05).

**Conclusion:**

Symptom association probability with an abnormal DeMeester score did not enhance the prediction of antireflux surgery outcome. These findings suggest that SAP should not be used in surgical decision-making in patients with objective evidence of reflux.

**Graphical Abstract:**

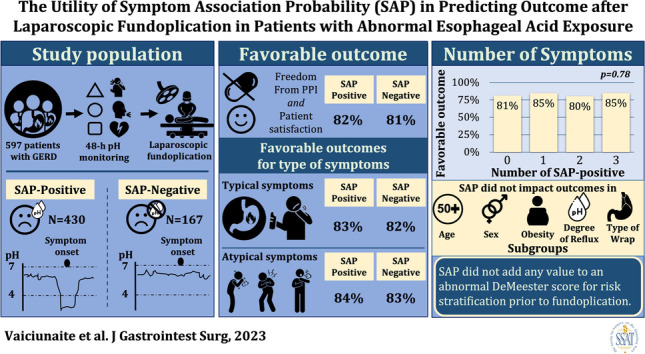

## Introduction

Antireflux surgery (ARS) has a high success rate for the management of gastroesophageal reflux disease (GERD), ranging from 84 to 96% in the appropriately selected patient.^1^ Confirmation that the patient’s symptoms are due to GERD is essential to appropriate patient selection.^2^ The predictability of success after any *ARS* relates directly to the degree of certainty that gastroesophageal reflux is the underlying cause of the patient’s symptoms. Esophageal pH monitoring has the highest sensitivity and specificity of the available tests to establish this causality. Consequently, an increased distal esophageal acid exposure is the strongest overall outcome predictor of a laparoscopic Nissen fundoplication. Studies have demonstrated that patients with an abnormal DeMeester score on pH monitoring are 5.4 times more likely to response to ARS.^3^ However, some clinicians have argued that the presence of pathological reflux provides no direct evidence that the patient’s symptoms are caused by acid reflux episodes.^4^ They have recommended that abnormal distal esophageal acid exposure should be interpreted in the setting of a temporal association between symptoms and reflux episodes.

Continuous pH monitoring provides the opportunity for patients to document symptom events throughout the recording period. These symptom events can be temporally correlated to acid reflux episodes to determine if there is a symptom–reflux association. Previous studies have represented this symptom–reflux association with a number of different symptom–reflux indices, but the three most commonly used are the symptom index (SI), the symptom sensitivity index (SSI), and the symptom association probability (SAP). The SI and SSI represent the percent of symptom–reflux-associated episodes, while the SAP represents the probability that the symptom–reflux association is not due to chance. Studies comparing these symptom–reflux indices have found that the SAP provides the least degree of day-to-day variability and is regarded as the most reliable.^5^

A positive symptom–reflux index signifies that the patient’s symptoms are caused by acid reflux episodes. Several studies have demonstrated that a positive symptom–reflux index is a predictor of symptomatic response to acid suppression therapy.^6,7^ Limited studies have suggested that a similar relationship may exist with antireflux surgery.^8^ Consequently, some symptom-oriented clinicians have argued that abnormal distal esophageal acid exposure should be interpreted in the context of SAP prior to surgery. However, there is paucity of data on whether assessing a symptom–reflux index adds any value in the surgical population. Therefore, we designed this study to determine the utility of SAP in predicting outcomes after primary Nissen or partial fundoplication in patients with abnormal distal esophageal acid exposure.

## Methods

### Study Population

This study was a retrospective review of prospectively collected data of patients who underwent preoperative pH monitoring followed by a primary Nissen or partial fundoplication at Allegheny Health Network hospitals (Pittsburgh, PA) between January 2015 and November 2021. The inclusion criteria were patients with a diagnosis of GERD, who were 18 years or older; with an abnormal distal esophageal acid exposure defined by DeMeester score >14.7 on 48-h pH monitoring; at least one documented SAP for up to 3 symptoms; underwent primary Nissen or partial fundoplication; and were assessed for postoperative satisfaction, freedom from proton pump inhibitors (PPI), and improvement in the GERD–Health-Related Quality of Life (GERD-HRQL) questionnaire. This study was evaluated and approved by the IRB of the Allegheny Health Network (IRB No. 2020-0687).

### GERD–Health-Related Quality of Life Measures

Patients completed the validated GERD–Health-Related Quality of Life (GERD-HRQL) questionnaire preoperatively and then again at 6 and 12 months postoperatively. The GERD-HRQL consists of 16 disease-specific severity questions scored from 0 to 5. Total score ranges from 0 to 80 with more severe symptoms indicated by a higher score. Additionally, a 17th question states “How satisfied are you with your present condition?” to assess overall patient satisfaction with the state of their GERD.^9^

### Preoperative Clinical and Objective Evaluation

All patients underwent a comprehensive clinical evaluation with a focus on their GERD symptoms and use of anti-secretory medications. In addition, preoperative assessment included esophagogastroduodenoscopy (EGD) and 48-h esophageal pH monitoring. EGD with biopsies allowed for the assessment of esophageal dilation, tertiary contractions, esophagitis, liquid or food retention, Barrett’s esophagus, and anatomical consideration such as a presence and size of a hiatal hernia, and Hill grade classification. During the EGD, a Bravo (Medtronic, Minneapolis, MN) esophageal pH monitoring capsule was placed 6cm above the gastroesophageal junction. Patients were asked to stop taking any anti-secretory medications 10 days prior to and for the duration of testing. Abnormal distal esophageal acid exposure was defined as a DeMeester score >14.7. Mild objective reflux was defined as a DeMeester score 14.7–20. Severe objective reflux was defined as a DeMeester score > 50.

### Assessment of Symptom Association Probability (SAP)

The Bravo esophageal pH monitoring system allows patients to report the occurrence of up to three of their most bothersome reflux symptoms. As distal esophageal pH is continuously recorded over 48 h, patients report each time they experience a symptom event. The SAP was calculated as previously described. The total test time was divided into consecutive 2-min periods. These 2-min periods were evaluated for the occurrence of gastroesophageal reflux episodes. A 2-min period was considered reflux-positive if there was a decrease in the distal esophageal pH below 4 for at least 5 consecutive seconds or a decrease in pH of more than 1 pH unit within a 5-s interval during the 2-min time period.^5^ Then all 2-min periods are assessed for patient-reported symptom occurrence. An episode was considered symptomatic if any of the up to three reported symptoms occurred during the 2-min period. Subsequently, the Fisher’s exact test was used to determine the probability that the association between symptomatic and reflux-positive 2-min periods was due to chance. A symptom was considered SAP-positive if this probability was ≥95%.^5,10^ A patient was considered SAP-positive if at least one symptom was SAP-positive.

### Outcomes and Definitions

Patient outcomes were evaluated at routine 6-month and 12-month postoperative visits. Favorable outcome was defined as both patient satisfaction and freedom from PPI at 1 year postoperatively. Additionally, patients were assessed for at least a 50% improvement in their GERD-HRQL questionnaire score from baseline to 1-year follow-up. For the purposes of analysis heartburn, regurgitation, and dysphagia were considered typical reflux symptoms; chest pain, cough, globus sensation, and throat clearing were considered atypical reflux symptoms; and abdominal pain and nausea were considered non-reflux symptoms.

### Statistical Analysis

The primary analysis was a comparison of baseline demographic and clinical characteristics as well as surgical outcomes between SAP-positive and SAP-negative patients. Sub-analysis was performed to determine if there was any difference in the number (0, 1, 2, or 3) of SAP-positive symptoms. An additional sub-analysis was performed to determine if SAP-positive symptom type (typical, atypical or non-reflux) resulted in a different favorable outcome rate than SAP-negative symptom type. A similar analysis was performed for each individual symptom. Finally, patients were divided into 14 subgroups on the basis of age, sex, BMI, preoperative GERD-HRQL score, mild objective reflux, severe objective reflux, and surgical procedure type. Multiple subgroup analyses were performed to determine if there was a specific population of patients whose favorable outcome rate differed between SAP-positive and SAP-negative groups. Values for continuous variables are expressed as either mean (SD) or median with an interquartile range when appropriate. Values for categorical variables are presented as frequency and percentage. Statistical analysis was performed by means of nonparametric Mann–Whitney *U* test, Wilcoxon signed-rank test, Kruskal–Wallis test, and Fisher’s exact test when appropriate. A *p* value < 0.05 was considered significant. Statistical analysis was performed using SAS software (SAS Institute Inc., Cary, NC).

## Results

A total of 597 patients underwent primary (89.1%) minimal invasive Nissen or (10.9%) partial fundoplication during this study period. Baseline demographic and clinical data are shown in Table 1. At a mean (SD) follow-up of 10.5 (8.0) months, the GERD-HRQL total score decreased from 36.2 (19.5) to 10.6 (13.5), with 74.7% achieving at least 50% total score improvement. Favorable outcome was achieved by 82% of patients, with 87.4% reporting satisfaction with the outcomes of the surgery and 91.7% remaining free from PPI use.

**Table 1:** Baseline demographic and clinical characteristics

Characteristic	Total patients (*N*=597)
Age, y, median (IQR)	59.0 (49–67)
Sex (female), *n* (%)	426 (71.4%)
BMI, kg/m^2^, median (IQR)	29.1 (26–33)
BMI >30, *n* (%)	262 (43.9%)
DeMeester score, median (IQR)	44.1 (29–62)
Mild objective reflux, *n* (%)	44 (7.4%)
Severe objective reflux, *n* (%)	243 (40.7%)
GERD-HRQL total score, median (IQR)	36.0 (21–51)
Fundoplication type, *n* (%)	
Partial	65 (10.9%)
Nissen	532 (89.1%)

*IQR* interquartile range, *BMI* body mass index

There were 430 (72.0%) patients with at least one SAP-positive symptom, and 167 (28.0%) patients were SAP-negative for all of their most bothersome symptoms during preoperative pH monitoring. Baseline demographic and clinical characteristics are compared between SAP-positive and SAP-negative patients as shown in Table 2. SAP-positive patients had a slightly higher BMI (29.3 (26–33) vs 28.4 (24–33), *p*=0.037), but the frequency of a BMI >30 was comparable between groups**.** Postoperatively, there was no difference in favorable outcome rates between SAP-positive and SAP-negative groups, with similar patient satisfaction and freedom from PPI rates (Table 3). Additionally, GERD-HRQL score improvement was similar.

**Table 2:** Comparison of baseline demographic and clinical characteristics between SAP groups

Characteristic	SAP-positive (*n*=430)	SAP-negative (*n*=167)	*p* value
Age, y, median (IQR)	58.0 (48–67)	61.0 (51–69)	0.063
Sex (female), *n* (%)	307 (71.4%)	119 (71.3%)	1.000
BMI, kg/m^2^, median (IQR)	29.3 (26–33)	28.4 (24–33)	0.037
BMI >30, *n* (%)	196 (45.6%)	66 (39.5%)	0.199
DeMeester score, median (IQR)	44.6 (32–62)	40.0 (28–65)	0.154
Mild objective reflux, *n* (%)	402 (93.5%)	151 (90.4%)	0.222
Severe objective reflux, *n* (%)	180 (41.9%)	63 (37.7%)	0.404
GERD-HRQL total score, median (IQR)	37.0 (23–51)	32.0 (18–51)	0.258
Fundoplication type, *n* (%)			
Partial	39 (9.1%)	26 (15.6%)	0.028
Nissen	391 (90.9%)	141 (84.4%)

**Table 3:** Comparison of surgical outcomes among SAP-positive and SAP-negative groups

Outcome, *n* (%)	SAP-positive (*n*=430)	SAP-negative (*n*=167)	*p* value
Favorable outcome	246 (82.8%)	87 (81.3%)	0.7673
Satisfaction	262 (87.9%)	92 (86.0%)	0.6123
Freedom from PPI	391 (92.0%)	149 (90.9%)	0.6218
50% GERD-HRQL improvement	174 (76.3%)	56 (70.0%)	0.2961

*GERD-HRQL* Gastroesophageal Reflux Disease–Health-Related Quality of Life, *PPI* proton pump inhibitors

### Impact of the Number of SAP-Positive Symptoms

**Fig. 1:**
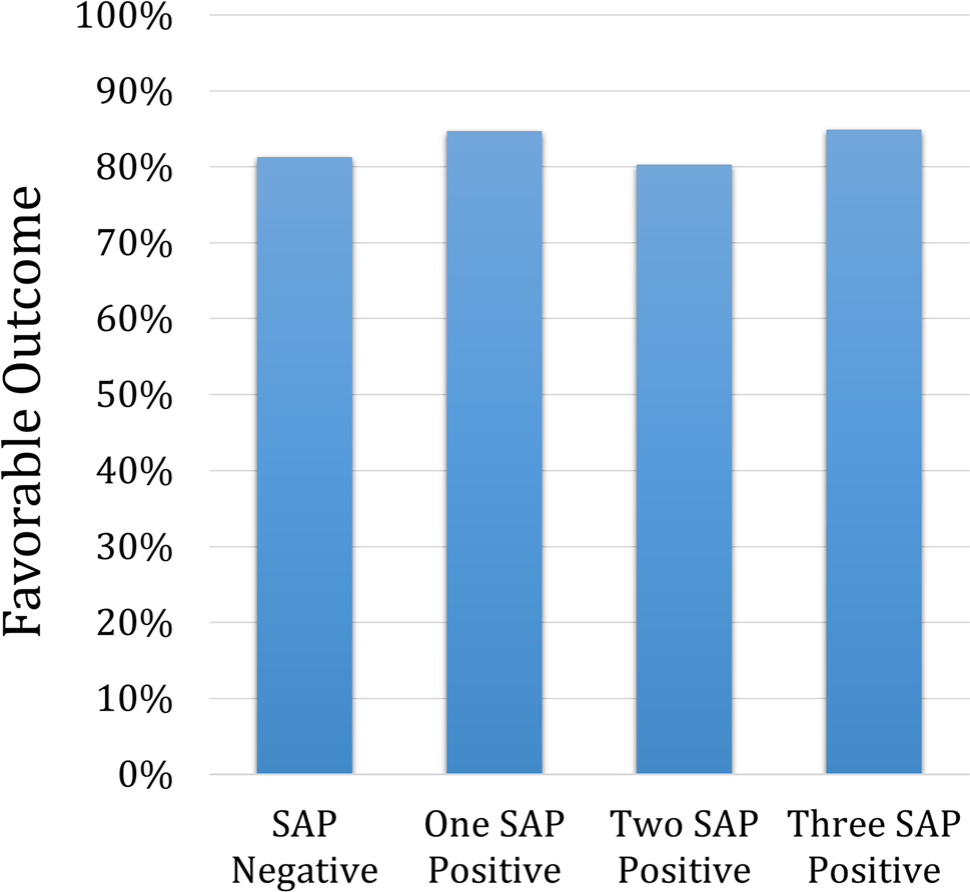
The comparison of the favorable outcome rate between patients with zero, one, two, or all three SAP-positive symptoms. There was no significant difference between groups (*p*=0.785)

There were 221 (37%) patients who had only one SAP-positive symptom, 164 (27.5%) with two SAP-positive symptoms, and 45 (7.5%) patients with all three symptoms SAP-positive. There was no difference in favorable outcome rates between patients who had no, one, two, or three SAP-positive symptoms (*p* = 0.7846) (Fig. 1).

### Typical and Atypical SAP-Positive Symptoms

The comparison of favorable outcome rates for SAP-positive vs negative typical, atypical, and non-reflux symptoms is shown in Table 4. There was no difference in outcome if a patient was SAP-positive for typical (*p* = 0.8731), atypical (*p* = 1.000), or non-reflux (*p*=0.3047) symptoms. In addition, the individual symptoms within those three groups were also all comparable (Table 5).

**Table 4:** Comparison of favorable outcome groups between SAP groups for symptom types

Symptom types, *n* (%)	SAP-positive	SAP-negative	*p* value
Typical symptoms	179 (83.3%)	113 (83.1%)	1.000
Atypical symptoms	101 (83.5%)	102 (83.6%)	1.000
Non-reflux symptoms	21 (72.4%)	22 (57.9%)	0.305

**Table 5:** Comparison of favorable outcome rates between SAP groups for individual symptoms

Individual symptoms, *n* (%)	SAP-positive	SAP-negative	*p* value
Heartburn	122 (83.0%)	94 (87.0%)	0.482
Chest pain	25 (83.3%)	31 (86.1%)	1.000
Regurgitation	75 (87.2%)	74 (73.1%)	0.526
Dysphagia	21 (80.8%)	33 (76.7%)	0.771
Cough	62 (81.6%)	71(85.5%)	0.527
Globus sensation	4 (100.0%)	4 (66.7%)	0.467
Throat clearing	22 (88.0%)	19 (86.4%)	1.000
Abdominal pain	10 (66.7%)	13 (72.2%)	1.000
Nausea	13 (92.9%)	18 (78.3%)	0.376

### The Value of SAP Across Demographic and Clinical Subgroups

**Fig. 2:**
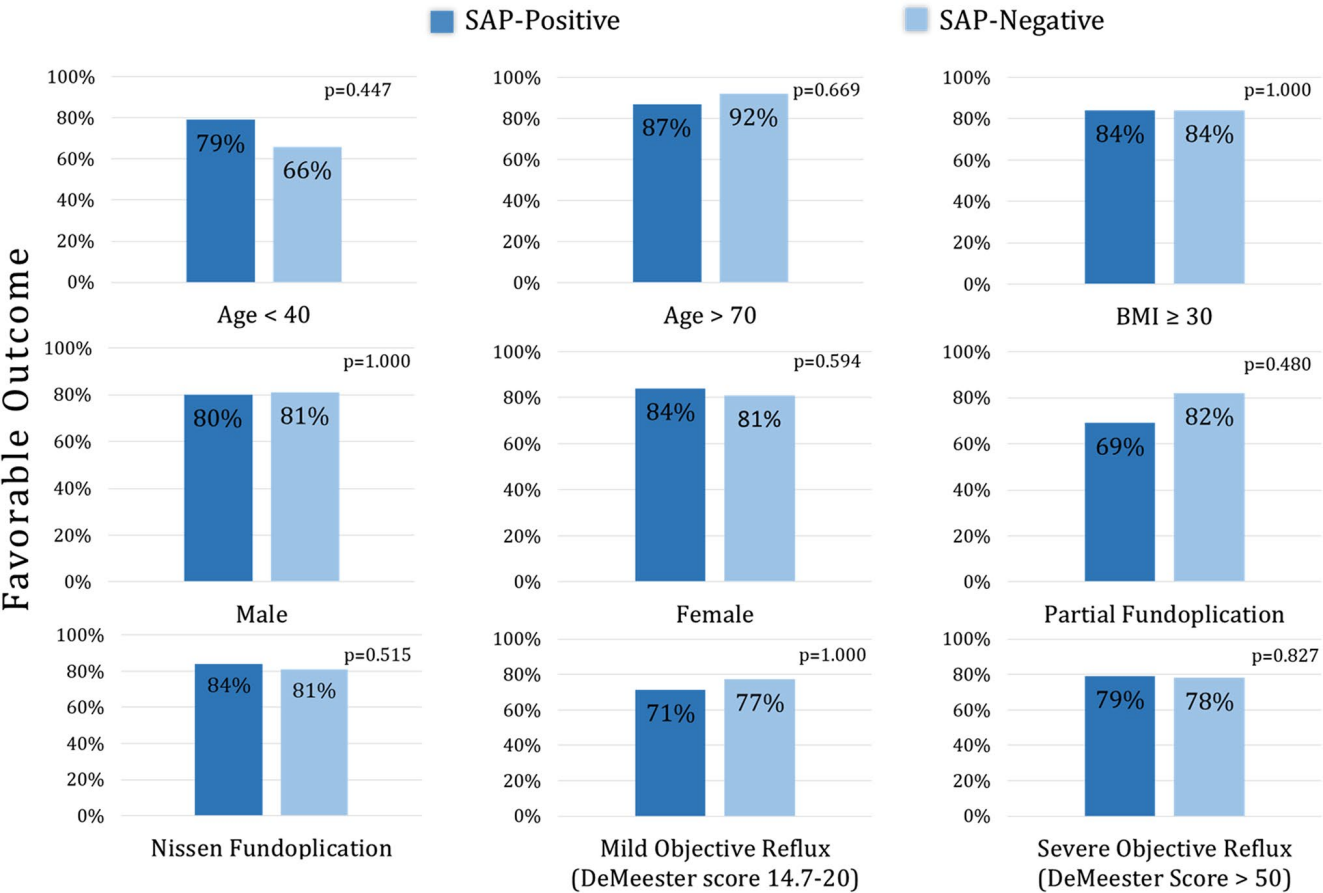
Favorable outcome was compared between SAP-positive and SAP-negative groups within nine subgroups based on demographics, severity of reflux, and type of fundoplication. Outcome was comparable in all groups

There were several subgroups examined for differences in favorable outcome rate between SAP-positive and SAP-negative patients (Fig. 2). There were 80 (13.4%) patients aged younger than 40. Favorable outcome in this subgroup was 76.0% and was similar between SAP groups (*p*=0.447). There were 105 (17.6%) patients aged 70 or older of which 91.0% achieved favorable outcome and was similar between SAP groups (*p*=0.669). The population had 426 (71.4%) female patients, 83.2% of which achieved favorable outcome, but SAP had no impact (*p*=0.594). Favorable outcome among male patients was 80.5% and was also similar between SAP groups (*p*=1.000). BMI was ≥ 30 in 262 (43.9%) patients, 84.5% of which achieved favorable outcome. SAP had no impact in this subgroup (*p*=1.000).

Objective reflux was mild in 44 patients (7.4%), 75.9% of which achieved favorable outcome, but SAP had no impact (*p*=1.000). Severe reflux was found in 243 (40.7%) patients, of which 79.1% had favorable outcome, and SAP had no impact (*p*=0.827). Finally, there were 532 (89.1%) patients who underwent Nissen fundoplication, of which 83.4% had favorable outcome, which was similar between SAP groups (*p*=0.515). Among those who underwent partial fundoplication, favorable outcome was 74.4% and similar between SAP groups (*p*=0.480).

## Discussion

The development of prolonged esophageal pH monitoring by Johnson and DeMeester in 1974 allowed objective documentation of reflux disease. Subsequent studies of antireflux surgery outcomes established an abnormal DeMeester score as the most impactful independent predictor of favorable outcome. These studies concluded that increased confidence in diagnosis led to confidence in outcome. Thus, pH monitoring became an essential test in the evaluation of a potential surgical patient with the clinical suspicion of GERD. However, some clinicians have questioned whether increased esophageal acid exposure was evidence that patients’ symptoms were due to reflux. A study of 50 patients who underwent acid perfusion testing and 24-h pH monitoring with an assessment of the symptom index (SI) found that almost half of patients with abnormal acid exposure had a negative SI.^11^ This led to an argument that abnormal acid exposure should be interpreted in the context of symptom–reflux association, assuming that a positive symptom–reflux index will better predict surgical outcome. In this study, we tested this assumption and found that in patients with abnormal distal esophageal acid exposure, surgical outcomes were comparable, regardless of whether SAP was positive or negative. Further, this index made no difference even when looking at the number of SAP-positive symptoms, type of symptoms, specific individual symptoms, or among several clinically relevant subgroups. Therefore, in a patient with an abnormal DeMeester score, SAP did not enhance the prediction of antireflux surgery outcome.

The intent of the present study was to test the assumption that interpreting abnormal esophageal acid exposure in context of a positive symptom–reflux index is important in the preoperative assessment. This assumption is rooted in studies demonstrating that SAP is a reliable predictor of response to PPIs. Taghavi et al. prospectively evaluated patient response to 1-week of high-dose omeprazole in 38 patients with heartburn predominant GERD.^7^ They found that SAP-positive patients were significantly more likely to have either complete symptom resolution or a 50% improvement in their symptom scores. However, they did not distinguish between patients with and without abnormal distal esophageal acid exposure. Similarly, a double-blind crossover study of 18 patients found that patients who were SI-positive had greater improvement in symptoms on omeprazole compared to placebo than SI-negative patients. However, all patients in this cohort had normal acid exposure.^6^ This is consistent with the findings of Aanen et al. who studied 74 patients who were divided into four SAP and objective pH-positive or pH-negative groups before a 2-week trial of 40-mg esomeprazole daily. They found that SAP-negative pH-negative patients did worse than every other group. However, they also found that among patients with abnormal distal esophageal acid exposure there was no difference in symptom improvement between SAP-positive and SAP-negative patients after PPIs, similar to our findings with ARS.^12^ These studies suggest that SAP may have a role in predicting outcomes, but the value is in patients without abnormal acid exposure. Similarly, a 5-year retrospective review of predictors of PPI therapy outcomes in 128 patients found that neither SAP nor SI based on acid reflux episodes were predictors of outcome on univariate analysis, but abnormal distal esophageal acid exposure and SAP based on impedance reflux episodes were independent predictors of favorable outcome on multivariate analysis.^13^ However, none of these studies demonstrated that SAP adds any value if the patient has abnormal distal esophageal acid exposure, which is similar to our findings in the surgical population.

The present study represents the largest volume evaluation of the symptom–reflux association in the surgical population to date. However, a limited number of smaller volume studies have similarly found that symptom–reflux association has no impact on surgical outcomes. Broaders et al. compared 5-year outcomes of Nissen fundoplication between 109 SAP-positive and 29 SAP-negative patients.^14^ They found comparable relief of reflux symptoms, reduction in PPI use, improvement in quality of life, reduction in acid exposure time, and improvement in esophagitis between SAP groups. Another study of 142 patients who underwent Nissen fundoplication similarly found comparable postoperative GERD-HRQL scores and symptom resolution between SAP-positive and SAP-negative patients.^15^ A study of 58 patients with medical refractory GERD who underwent ARS also found that pre-surgery SAP was not associated with response to antireflux surgery.^16^ These studies suggest that SAP is independent of surgical outcome; however, there are some studies that disagree. One study of 56 patients who underwent ARS and impedance–pH testing found that distal esophageal acid exposure was an independent predictor of favorable outcome (*p*<0.004) and that impedance-based, but not acid-based, positive SI trended towards predicting outcome (*p*=0.050).^17^ This finding is similar to the medical literature suggesting the symptom–reflux association has a role in the assessment of non-acid reflux. A study by Diaz et al. prospectively assessed the impact of SAP and SI on outcome of 79 patients with abnormal distal esophageal acid exposure and found that SAP predicted subjective patient-reported improvement at a mean of follow-up of 8.3 ± 0.7 months.^8^ Similarly, a single-center study of 33 patients who underwent Nissen fundoplication found that a positive SAP on pH impedance was the only predictor of successful outcome at a mean of 41.3 (range 7–102.2) months after surgery.^18^ The inconsistent results in these studies may be due to limited sample size, limited and inconsistent follow-up, highly subjective definitions of outcome, and the use of impedance rather than pH monitoring. Based on all the available data in the surgical population, there is insufficient quality evidence that SAP adds any additional value when a patient has abnormal distal esophageal acid exposure. However, there is strong evidence that distal esophageal acid exposure is independently associated with outcome. Therefore, patients with objective evidence of reflux should be considered for antireflux surgery, regardless of symptom association indices.

Nissen fundoplication provides excellent outcomes for patients with typical reflux symptoms.^2^ However, atypical symptoms as seen with laryngopharyngeal reflux (LPR) are highly non-specific and can therefore be a challenge to both diagnose and treat.^19^ Studies have demonstrated that pH monitoring has limited sensitivity and specificity in patients with LPR. Therefore, we compared typical and atypical symptoms to determine if SAP could aid in identifying patients with atypical symptoms who are more likely to respond to ARS. However, outcomes were similar for both typical and atypical symptoms as well as for all individual symptoms. Previous studies have found similar results. Chin et al. compared fundoplication outcomes between patients who were SI-positive for typical symptoms (*n*=104), SI-positive for atypical symptoms (*n*=28), and SI-negative (*n*=23). They found comparable heartburn scores, dysphagia scores, symptoms of bloating and belching, and willingness to repeat surgery between SI groups at 1-year after fundoplication.^20^ Choski et al. also found similar outcomes in their study of SAP in 58 patients who underwent ARS for typical (58.6%) and atypical (41.4%) reflux symptoms. They found that despite a population with a comparable number of SAP-positive typical and SAP-positive atypical symptoms, SAP did not predict outcome.^16^ In contrast, Hersh et al. studied 53 patients who underwent pH monitoring for cough and found that SAP was an independent predictor of durable symptom improvement 3 years after antireflux therapy.^21^ However, the inconsistency of this result is likely due to the study design, which included patients with prior antireflux surgery and combined outcomes for both medical and surgical management of GERD. Additionally, only 47.2% of patients had abnormal distal esophageal acid exposure, a very low percentage for a surgical population. The most robust studies assessing typical and atypical symptoms agree that SAP does not impact surgical outcome.

In order to thoroughly examine the value of SAP in patients with an abnormal DeMeester score, we examined favorable outcome in multiple subgroups. SAP had no impact within subgroups based on age, sex, or BMI demographics. The greatest potential for SAP to make a clinically significant impact was in the population of patients with borderline abnormal DeMeester scores, as they represent the least certainty that symptoms are due to reflux*.* However, when we examined this subgroup, we found that SAP had no impact on outcome. One potential confounder is the reliability of SAP at lower pH. Slaughter et al. used Monte Carlo simulation to investigate factors affecting whether SAP was positive due to a causal relationship or chance. They found that SAP was only better than chance in patients with higher acid reflux rates, concluding that SAP may be most dependable in patients with high reflux rates.^22^ Therefore, we evaluated a subgroup of patients with a DeMeester score >50 but still found that SAP had no impact on surgical outcomes.

The SAP may be regarded as the most reliable symptom–reflux index, but it is not without its limitations. A fundamental limitation of all symptom–reflux indices is that they introduce the subjectivity of patient symptom reports into the objective measure of reflux. Aanen demonstrated that this factor affects the reproducibility of SAP. They found the number of reflux episodes were highly reproducible between tests with a concordance of 0.92, but the number of symptom episodes was not reproducible with a concordance of 0.75 (*p*=0.07), suggesting that variability in SAP is more due to inconsistent symptom reports than reflux episodes.^12^ A similar conclusion was reached in a prospective cross-sectional study of 21 patients with chronic cough as their most bothersome GERD symptom. They synchronized an acoustic recording system with 24-h ambulatory pH impedance monitoring and compared patient-reported cough symptoms to acoustically recorded coughs and found that patients did not report 71–91% of their audible cough events.^23^ This finding suggests that the reliance on the accuracy of patient symptom reports is a major limitation of symptom–reflux indices and may partially explain why in our cohort of patients with abnormal DeMeester score SAP groups were comparable in all analyses.

We acknowledge certain limitations of this study, including its retrospective nature. Patients were not randomized to Nissen or partial fundoplication. However, when we assessed the outcomes in the partial fundoplication subgroup, results were similar to the Nissen fundoplication subgroup. The purpose of this study was to assess if SAP added value to an abnormal DeMeester score on 48-h Bravo pH monitoring. As a result, patients who underwent fundoplication based on multichannel impedance pH testing were excluded. These patients were more likely to have laryngopharyngeal reflux. Further research on the impact of SAP on surgical outcomes in patients with a normal DeMeester score and/or those who underwent impedance testing is necessary to fully understand the relationship between SAP and surgical outcome. Therefore, the results of this study should be applied specifically to patients with abnormal distal esophageal acid exposure.

There have been several consensus papers aimed at providing guidelines for the diagnosis and assessment of GERD. The Montreal consensus has a symptom-oriented approach, arguing that GERD is a set of syndromes, which can be subdivided based on whether or not there are objective findings.^24^ However, this consensus does not directly comment on the utility of symptom–reflux association indices in the assessment of GERD. The Lyon consensus on the modern diagnosis of GERD, on the other hand, suggests that symptom–reflux indices are predictive of surgical outcome.^25^ However, this statement appears to come from the discussions of studies demonstrating a relationship between different symptom association indices and response to PPIs.^6,7,12,13^ However, these studies suggest that there may be a relationship between surgical outcomes and SAP in patients without abnormal distal esophageal acid exposure or with impedance-based SAP. Importantly, these study designs focused on either a different population or different modality than we assessed. As understanding of the relationship between symptom–reflux indices and outcome increases, a more nuanced statement may be warranted in future consensus meetings.

## Conclusion

The growing body of evidence that symptom–reflux indices are associated with antireflux medical therapy outcomes has encouraged some clinicians to stress the importance of interpreting abnormal distal esophageal acid exposure in the context of the SAP. In this study we tested this assumption by comparing outcomes between SAP-positive and SAP-negative patients with objective acid reflux and found that outcomes were comparable. Additionally, it made no difference how many of the patients symptoms were SAP-positive, what type of reflux symptoms the patient had or what the specific symptom was. Furthermore, these findings held true in subgroups based on age, sex, symptom severity, reflux severity, or type of fundoplication. Based on the findings of the current study, we recommend that if a patient has abnormal distal esophageal acid exposure, they should be considered for antireflux surgery, regardless of their symptom–reflux association indices.

## Data Availability

The data used during this study are available from the corresponding author upon reasonable request.

## References

[1] Eubanks TR, Omelanczuk P, Richards C, Pohl D, Pellegrini CA (2000). Outcomes of laparoscopic antireflux procedures. The American journal of surgery..

[2] Peters JH, DeMeester TR, Crookes P, Oberg S, de Vos SM, Hagen JA (1998). The treatment of gastroesophageal reflux disease with laparoscopic Nissen fundoplication: prospective evaluation of 100 patients with" typical" symptoms. Annals of surgery..

[3] Campos GM, Peters JH, DeMeester TR, Öberg S, Crookes PF, Tan S (1999). Multivariate analysis of factors predicting outcome after laparoscopic Nissen fundoplication. Journal of Gastrointestinal Surgery..

[4] Bredenoord A, Weusten B, Smout A (2005). Symptom association analysis in ambulatory gastro-oesophageal reflux monitoring. Gut..

[5] Weusten BL, Roelofs JM, Akkermans LM, Van Berge-Henegouwen GP, Smout AP (1994). The symptom-association probability: an improved method for symptom analysis of 24-hour esophageal pH data. Gastroenterology..

[6] Watson R, Tham T, Johnston B, McDougall N (1997). Double blind cross-over placebo controlled study of omeprazole in the treatment of patients with reflux symptoms and physiological levels of acid reflux--the" sensitive oesophagus". Gut..

[7] Taghavi SA, Ghasedi M, Saberi-Firoozi M, Alizadeh-Naeeni M, Bagheri-Lankarani K, Kaviani MJ (2005). Symptom association probability and symptom sensitivity index: preferable but still suboptimal predictors of response to high dose omeprazole. Gut..

[8] Diaz S, Aymerich R, Clouse RE, et al. The symptom association probability (SAP) is superior to the symptom index (SI) for attributing symptoms to gastroesophageal reflux: validation using outcome from laparoscopic antireflux surgery (LARS). Gastroenterology. 2002;122:A75.

[9] Velanovich V (2007). The development of the GERD-HRQL symptom severity instrument. Diseases of the Esophagus..

[10] Kamal AN, Clarke JO, Oors JM, Smout AJ, Bredenoord AJ (2020). The role of symptom association analysis in gastroesophageal reflux testing. Official journal of the American College of Gastroenterology|. ACG..

[11] Howard P, Maher L, Pryde A, Heading R (1991). Symptomatic gastro-oesophageal reflux, abnormal oesophageal acid exposure, and mucosal acid sensitivity are three separate, though related, aspects of gastro-oesophageal reflux disease. Gut..

[12] Aanen MC, Weusten BL, Numans ME, de Wit NJ, Samsom M, Smout AJ (2008). Effect of proton-pump inhibitor treatment on symptoms and quality of life in GERD patients depends on the symptom-reflux association. Journal of clinical gastroenterology..

[13] Patel A, Sayuk GS, Gyawali CP (2015). Parameters on esophageal pH-impedance monitoring that predict outcomes of patients with gastroesophageal reflux disease. Clinical Gastroenterology and Hepatology..

[14] Broeders J, Draaisma W, Bredenoord A, Smout A, Broeders I, Gooszen H (2011). Impact of symptom–reflux association analysis on long-term outcome after Nissen fundoplication. Journal of British Surgery..

[15] Jalilvand AD, Martin Del Campo SE, Hazey JW, Perry KA. Impact of symptom association probability on outcomes of laparoscopic Nissen fundoplication. (2017). https://www.asc-abstracts.org/abs2017/16-20-impact-of-symptom-association-probability-on-outcomes-of-laparoscopic-nissen-fundoplication/.

[16] Choksi Y, Slaughter J, Sharda R, Higginbotham T, Lal P, Vaezi M (2018). Symptom association probability does not reliably distinguish functional heartburn from reflux hypersensitivity. Alimentary Pharmacology & Therapeutics..

[17] Patel A, Aadam AA, Sayuk GS, Gyawali CP (2012). Su1095 Reflux Exposure Time on pH-Impedance Testing Predicts Symptom Improvement After Antireflux Surgery (ARS) Better Than Number of Reflux Events. Gastroenterology..

[18] Desjardin M, Luc G, Collet D, Zerbib F (2016). 24-hour pH-impedance monitoring on therapy to select patients with refractory reflux symptoms for antireflux surgery. A single center retrospective study. Neurogastroenterology & Motility..

[19] Ayazi S, Hagen JA, Zehetner J, Lilley M, Wali P, Augustin F (2010). Loss of alkalization in proximal esophagus: a new diagnostic paradigm for patients with laryngopharyngeal reflux. Journal of Gastrointestinal Surgery..

[20] Chin K-F, Myers J, Jamieson G, Devitt P (2008). Symptoms experienced during 24-h pH monitoring and their relationship to outcome after laparoscopic total fundoplication. Diseases of the Esophagus..

[21] Hersh MJ, Sayuk GS, Gyawali CP (2010). Long-term therapeutic outcome of patients undergoing ambulatory pH monitoring for chronic unexplained cough. Journal of clinical gastroenterology..

[22] Slaughter JC, Goutte M, Rymer JA, Oranu AC, Schneider JA, Garrett CG (2011). Caution about overinterpretation of symptom indexes in reflux monitoring for refractory gastroesophageal reflux disease. Clinical Gastroenterology and Hepatology..

[23] Kavitt RT, Higginbotham T, Slaughter JC, Patel D, Yuksel ES, Lominadze Z (2012). Symptom reports are not reliable during ambulatory reflux monitoring. Official journal of the American College of Gastroenterology|. ACG..

[24] Vakil N, Van Zanten SV, Kahrilas P, Dent J, Jones R, Group GC (2006). The Montreal definition and classification of gastroesophageal reflux disease: a global evidence-based consensus. Official journal of the American College of Gastroenterology|. ACG..

[25] Gyawali CP, Kahrilas PJ, Savarino E, Zerbib F, Mion F, Smout AJ (2018). Modern diagnosis of GERD: the Lyon Consensus. Gut..

